# Obsessive and compulsive symptoms in elderly: A litterature review

**DOI:** 10.1192/j.eurpsy.2023.1965

**Published:** 2023-07-19

**Authors:** N. Kouki, A. Maamri, M. Hsayri, N. Kouki, A. Hajri, H. Zalila

**Affiliations:** 1 Outpatient department, Razi hospital, Manouba; 2 Neurology department, charles nicolle, tunis, Tunisia

## Abstract

**Introduction:**

Obsessive compulsive symptoms may occur at any age. It was studied extensively in the youth contratry to the elderly that usually suffer for other health issues leading to misdiagnosis.

**Objectives:**

We aim through this study to discover the particularities of OCD in elderly .

**Methods:**

Our literature review was based on the PubMed interface and adapted for 2 databases: Science Direct and Google Scholar using the following combination ( obsessive compulsive disorder [MeSH terms]) OR (OCD [MeSH terms]) AND (elderly [MeSH terms]) OR (dementia [MeSH terms]).

**Results:**

Our review revealed 39 articles from which we selected 4 articles.

We found that in aged adults over than 50 years experience mostly somatic symptoms, religiosity, and moral scrupulosity as obsessive thoughts.

We also found that OCD can occur as a primary disorder in older women, whereas in men it either persists from, younger years or arises in the context of another psychiatric or medical disorder

The relationship between Obsessional illness, brain mechanisms and Cognitive disorders are not fully understood.

Indeed, we noted a relative Impairment in executive function in older adults with OCD stressing the link with cognitive impairment. Moreover, Obessive compulsive symptoms may worsen cognitive functioning .

**Conclusions:**

Obsessive compulsive disorder presence in the elderly may be described as a primary health condition or be related to organic mental health issues in particular dementia. The physiopathology remains unclear therefore further studies are needed for better understanding and management.

**Disclosure of Interest:**

None Declared

